# Screening for potential genes associated with bone overgrowth after mid-shaft femur fracture in a rat model

**DOI:** 10.1186/s13018-017-0510-6

**Published:** 2017-01-17

**Authors:** Chibing Liu, Yanting Liu, Weizhong Zhang, Xiuxin Liu

**Affiliations:** 1 The Second Hospital of Jilin University, Ziqiang St 218 Nan Guan District, Changchun, 130041 China; 2 Jilin University, Changchun, 130012 China; 3 China-Japan Union Hospital of Jilin University, Changchun, Jilin 130000 China; 4 The Six Affiliated Hospital of Xinjiang Medical University Medical Examination Center, Autonomous Region, Five Star South Road, No. 39, Urumqi City, Xinjiang Uygur 830002 China

**Keywords:** Femoral fracture, Overgrowth, Protein-protein interaction network, Module

## Abstract

**Background:**

We investigated the underlying molecular mechanisms of bone overgrowth after femoral fracture by using high-throughput bioinformatics approaches.

**Methods:**

The gene expression profile of GSE3298 (accession number) was obtained from the Gene Expression Omnibus database. Sixteen femoral growth plate samples, including nine samples without fracture and seven fracture samples for seven time points, were used for analysis. The Limma package was applied to identify differentially expressed genes (DEGs) between fractured and intact samples. The DAVID online tool was used for Gene ontology functional and pathway enrichment analysis. A protein-protein interaction (PPI) network established by String software was used to identify interactions between significant DEGs, and network modules were detected using plug-in MCODE. Additionally, a transcription regulatory network was constructed based on the ENCODE Project and PPI network.

**Results:**

A total of 680 DEGs were screened in fractured femoral growth plate samples compared with controls, including 238 up- and 442 down-regulated genes. These DEGs were significantly involved in the calcium signaling pathway and cancer pathway. A PPI network was constructed with 167 nodes and 233 edges, and module analysis demonstrated that *CCL2*, *CSF2*, *NOS2*, and *DLC1* may stimulate bone overgrowth after femoral fracture via anti-apoptosis-related functions. A transcription regulatory network was constructed with 387 interacting pairs, and overlapping nodes were significantly enriched in intracellular signaling cascade and regulation of cell proliferation, among others.

**Conclusions:**

Bone overgrowth was associated with changes in the expression of identified DEGs such as *CCL2*, *NOS2*, *CSF2*, and *DLC1* in the femoral head. They may be important in regulating bone overgrowth via the anti-apoptosis of osteoblasts.

**Electronic supplementary material:**

The online version of this article (doi:10.1186/s13018-017-0510-6) contains supplementary material, which is available to authorized users.

## Background

Femoral fracture, which is one of the most commonly occurring fractures during childhood, always results from casual falls, motor vehicle accidents, or sporting accidents [1]. Treatment of femoral fracture typically includes open-reduction, traction, and internal fixation. However, pediatric femoral fracture often results in the stimulation of bone overgrowth, particularly in children younger than 12 years [2, 3]. Overgrowth is described as a universal phenomenon in patients with femoral shaft fractures and can elongate the lower limb by nearly 9 mm [4] or 11 mm [5]. It is crucial to explore the underlying molecular mechanism of bone overgrowth associated with femoral fracture.

In recent years, numerous studies have investigated the molecular mechanism of bone overgrowth after femoral fracture [6–8]. Bone homeostasis is thought to be maintained by a balance between bone formation by osteoblasts and bone resorption by osteoclasts in the growth plate. Various proteins such as β-catenin and triggering receptor expressed by myeloid cells-2 interact with each other by controlling the rate of osteoclastogenesis and further regulating bone homeostasis [9]. Several other factors were also found to be involved in this process. For instance, lipoprotein receptor-related protein 4 (LRP4) was found to be associated with the inhibitory function of sclerostin which is secreted by osteocytes and inhibits bone formation [6]. Additionally, the Wnt1/β-catenin signaling pathway is crucial for embryonic and bone homeostasis [10–12], and LRP4 may increase sclerostin secretion through Wnt1/β-catenin signaling [7]. Moreover, fibroblast growth factor receptor (FGFR) is also a critical gene in bone overgrowth and participates in FGFR3 signaling, further affecting chondrocyte proliferation [8]. Another report showed that osteocrin is highly expressed in osteoblasts and interacts with C-type natriuretic peptide receptors to modulate the action of the natriuretic system during bone elongation [13]. Therefore, expression changes of such related genes in cells after femoral fracture may provide insight into the physiological mechanisms of bone overgrowth.

In the past few years, DNA microarray technology has been increasingly utilized to comprehensively test for changes in the messenger RNA (mRNA) expression of genes and search for evidence of overgrowth after mid-femoral fracture [3, 14]. However, the potential molecular mechanism of bone overgrowth after mid-shaft femur fracture also remains unclear. The aim of this study was to explore potentially important genes associated with bone overgrowth after femoral fracture and clarify this phenomenon using high-throughput bioinformatics methods.

## Methods

### Data source

The gene expression profile of GSE3298 [3, 14], which describes mRNA expression in the rat proximal femoral growth plate after mid-shaft fracture, was derived from the Gene Expression Omnibus (GEO, http://ncbi.nlm.nih.gov/geo/) database based on the GPL1355 Affy metrix Rat Genome 230 2.0 Array platform (Santa Clara, CA, USA). A total of 16 femoral growth plate samples were used for analysis, including nine samples without fracture and seven fracture samples for seven time points: 1 day, 3 days, 1 week, 2 weeks, 3 weeks, 4 weeks, and 6 weeks, after mid-shaft fracture.

### Data preprocessing and differentially expressed genes (DEGs) screening

Each sample in the obtained dataset had a probe ID, which was converted into the corresponding gene name. Multiple probe IDs targeting the same gene were averaged as the gene expression value. After expression values were log_2_ transformed, quantile normalization was carried out [15]. The Limma package (http://www.bioconductor.org/packages/release/bioc/html/limma.html) [16] in R language was used to screen DEGs between intact and fractured samples. Gene expression differences were assessed using Student’s *t* test, and expression changes were considered to be significant when by *P* < 0.05.

### Functional annotation and pathway enrichment of DEGs

Functional annotation of genes was carried out using the Database for Annotation, Visualization, and Integrated Discovery (DAVID, http://david.abcc.ncifcrf.gov/) [17]. Gene ontology (GO, http://geneontology.org/) annotation and pathway enrichment analysis were performed to derive all associated functions with their enrichment scores and *P* values. Fisher’s exact test was used to evaluate the differences between the intact and fractured femora. Only results showing enrichment scores of more than 2 and *P* values <0.05 were considered to be statistically significant.

### Construction of protein-protein interaction (PPI) network

The PPI is thought to be important for understanding the potential functions of a certain protein. The up- and down-regulated genes identified as described above were respectively mapped to the Search Tool for the Retrieval of Interacting Genes (STRING, http://string-db.org/) software which is commonly used to predict PPI pairs [18]. The PPI network was constructed with interesting PPI pairs and visualized by Cytoscape 2.8 (http://cytoscape.org/) [19].

### Module detection

Molecular complex detection (MCODE) [20] is a clustering algorithm used to identify molecular complexes in the PPI network. Degree ≥2 and k-score ≥2 were selected as cutoff criteria. Next, Bingo [21] was applied to perform GO functional enrichment analysis with a threshold of adjusted *P* value <0.05, with multiple test adjustment conducted as described by Benjamini-Hochberg.

### Transcription regulatory network

The ENCODE (ENCyclopedia of DNA Elements) Project is designed to identify all functional components in the human genome sequence, including protein-coding genes, non-protein-coding genes, sequences that mediate chromosome structure and dynamics, and transcriptional regulatory elements [22]. Based on transcription factor information determined using ENCODE and the PPI network, a transcription regulatory network was constructed with interacting pairs using Cytoscape. Additionally, overlapping nodes in the network with node degrees of >2 were further analyzed for functional enrichment using DAVID. Only the results showing *P* values <0.05 were considered to be statistically significant.

## Results

### Identification of DEGs

After data preprocessing and quantile normalization, the gene expression profile of GSE3298 was used to screen for DEGs in the proximal femoral growth plate between intact and fractured samples. A total of 680 DEGs were screened out with *P* < 0.05, including 238 up- and 442 down-regulated genes (Additional file 1: Table S1).

### Functional analysis and pathway enrichment for DEGs

To evaluate DEG functions, GO and pathway analysis were performed for up-regulated and down-regulated genes, respectively. As shown in Table 1, up-regulated genes were mainly enriched in seven GO terms including cell fraction, response to organic substance, and response to wounding, among others, while down-regulated genes mainly function in the extracellular region, vesicles, cytoplasmic vesicles, and membrane-bound vesicles, among others. Additionally, up-regulated genes were found to be mainly enriched in six pathways such as the calcium signaling pathway (*P* = 0.001) and neuroactive ligand-receptor interactions (*P* = 0.001), while the 442 down-regulated DEGs were significantly enriched in four pathways, including pathways in cancer (*P* = 0.008), calcium signaling pathway (*P* = 0.016), hedgehog signaling pathway (*P* = 0.025), and MAPK signaling pathway (*P* = 0.028) (Table 2). These results suggest that perturbations in genes involved in these functions and/or pathways are associated with bone overgrowth following femoral fracture.

**Table 1:** Gene ontology (GO) analysis for differentially expressed genes (DEGs)

GO-ID	Description	Counts	*P* value
Up-regulated genes			
GO:0009611	Response to wounding	23	3.65E−09
GO:0006952	Defense response	20	1.82E−07
GO:0006954	Inflammatory response	15	2.29E−07
GO:0010033	Response to organic substance	29	2.52E−06
GO:0000267	Cell fraction	30	6.97E−06
GO:0042330	Taxis	9	5.29E−06
GO:0006935	Chemotaxis	9	5.29E−06
Down-regulated genes			
GO:0019935	Cyclic-nucleotide-mediated signaling	11	3.49E−05
GO:0031410	Cytoplasmic vesicle	27	3.23E−04
GO:0051046	Regulation of secretion	16	2.58E−04
GO:0031982	Vesicle	28	3.44E−04
GO:0019932	Second-messenger-mediated signaling	13	2.80E−04
GO:0007187	G-protein signaling, coupled to cyclic nucleotide second messenger	9	3.04E−04
GO:0031988	Membrane-bounded vesicle	25	4.69E−04
GO:0016023	Cytoplasmic membrane-bounded vesicle	24	6.11E−04
GO:0044421	Extracellular region part	29	6.64E−04

**Table 2:** Pathway enrichment for differentially expressed genes (DEGs)

Term	Count	*P* value
Up DEGs		
rno04020: Calcium signaling pathway	10	0.001065
rno04080: Neuroactive ligand-receptor interaction	12	0.001143
rno04610: Complement and coagulation cascades	6	0.003097
rno04062: Chemokine signaling pathway	7	0.034971
rno05322: Systemic lupus erythematosus	5	0.039347
rno04620: Toll-like receptor signaling pathway	5	0.039347
Down DEGs		
rno05200: Pathways in cancer	15	0.008138
rno04020: Calcium signaling pathway	10	0.016627
rno04340: Hedgehog signaling pathway	5	0.025443
rno04010: MAPK signaling pathway	12	0.028372

### Construction of PPI network

**Fig. 1:**
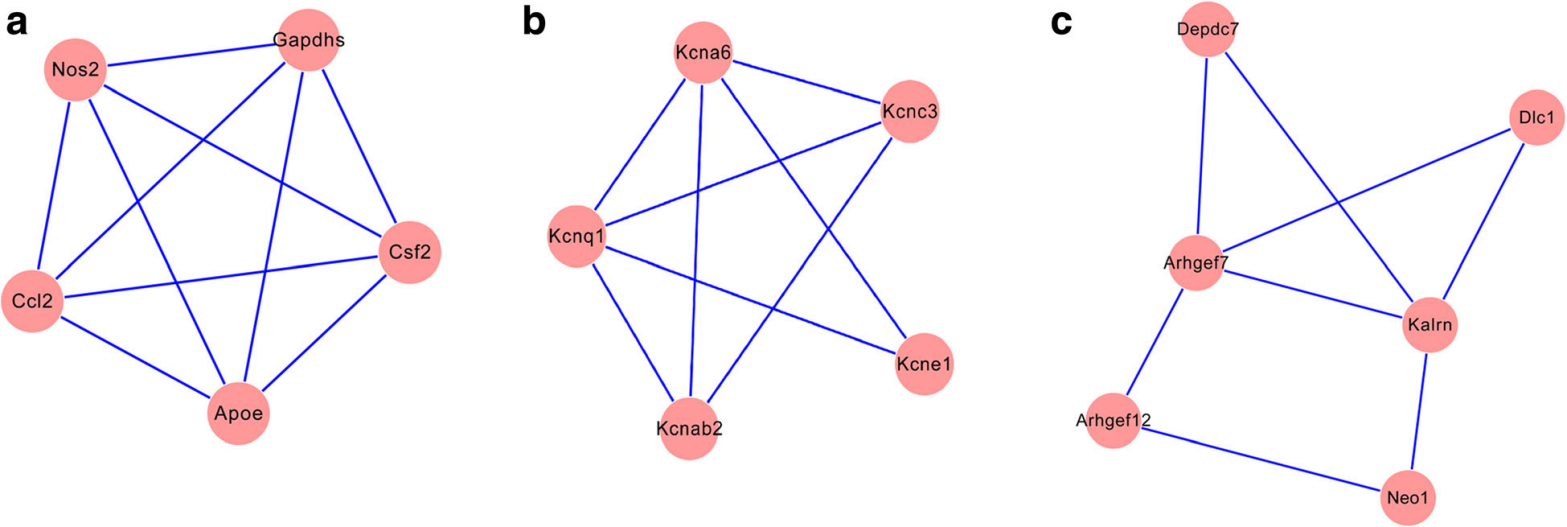
Protein-protein interaction network for differentially expressed genes (DEGs). *Red circles* indicate protein products of significant DEGs; *blue lines* indicate interaction between different proteins

To build the PPI network, significant protein interactions were predicted; the results are displayed in Additional file 2: Table S2. Based on these interaction pairs, a PPI network was constructed with 167 nodes and 233 edges (Fig. 1). Among all nodes, three DEGs showed relatively higher degrees, including chemokine (C–C motif) ligand 2 (*CCL2*), nitric oxide synthase 2 (*NOS2*), and colony-stimulating factor 2 (*CSF2*). Moreover, GO analysis suggested that up-regulated *CCL2* participated in the chemokine signaling pathway, and down-regulated *NOS2* involved cancer and calcium signaling pathways. In addition, down-regulated *CSF2* was mainly enriched in the hematopoietic cell lineage pathway.

### Significant module analysis

**Fig. 2:**
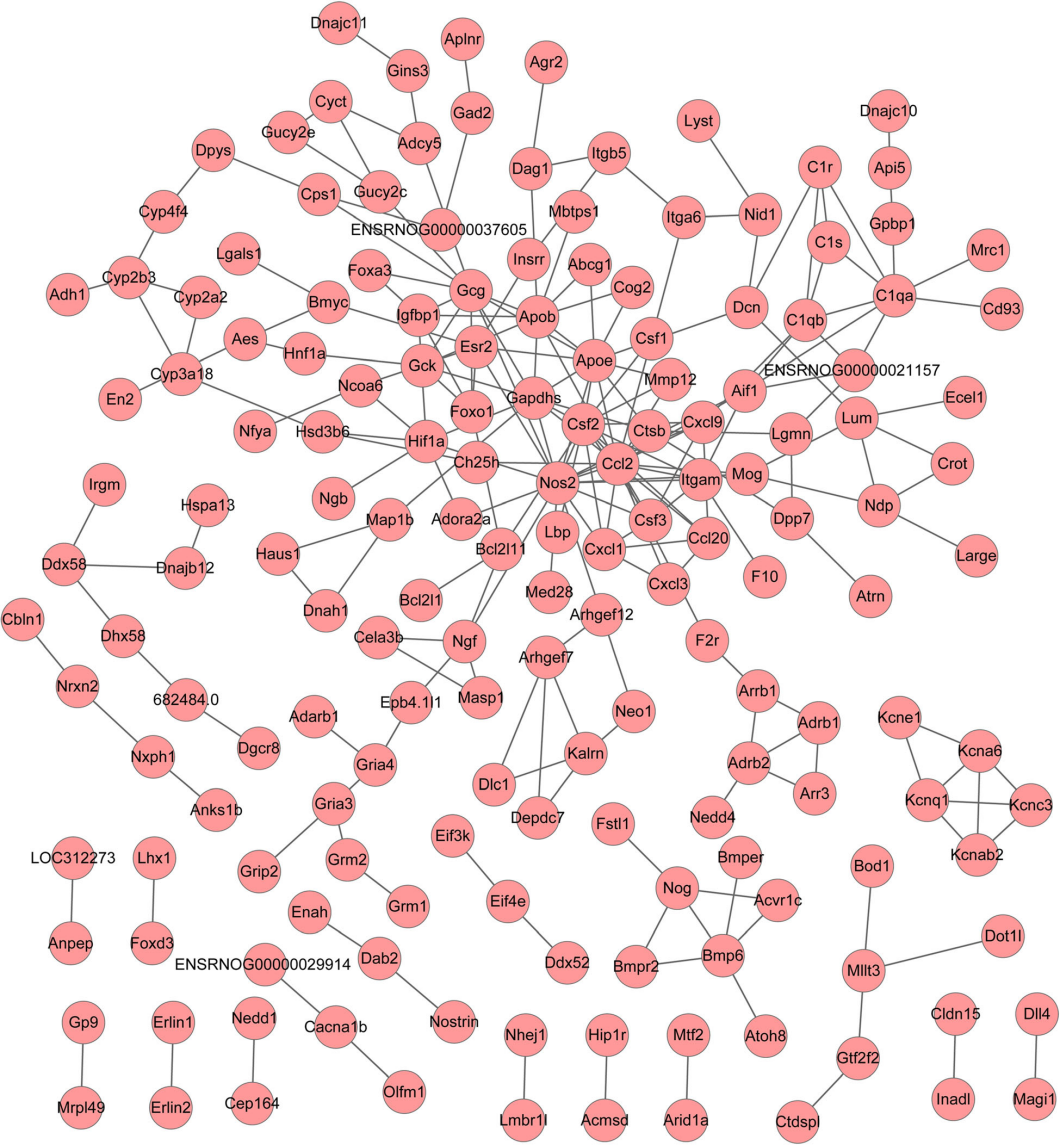
Three significant modules identified from PPI network. *Red circles* indicate protein products of differentially expressed genes; *blue lines* indicate interaction between different proteins

The PPI network was processed by MCODE and a total of 3 significant modules were obtained (Fig. 2). Module A contained one up-regulated DEG (*CCL2*) and four down-regulated DEGs (*NOS2*, *CSF2*, *APOE*, *GAPDHS*) . Five down-regulated DEGs made up module B, including potassium voltage-gated channel, shaker-related subfamily, member 6 (*KCNA6*), potassium voltage-gated channel, KQT-like subfamily, member 1 (*KCNQ1*), potassium voltage-gated channel, Shaw-related subfamily, member 3 (*KCNC3*), potassium voltage-gated channel, shaker-related subfamily, beta member 2 (*KCNAB2*), and potassium voltage-gated channel, Isk-related family, member 1 (*KCNE1*). Moreover, module C was constituted by one up-regulated DEGDEP domain containing seven (*DEPDC7*) and five down-regulated DEGs including Rho guanine nucleotide exchange factor 7 (*ARHGEF7*), Rho guanine nucleotide exchange factor 12 (*ARHGEF12*), deleted in liver cancer 1 (*DLC1*), kalirin, RhoGEF kinase (*KALRN*), and neogenin 1 (*NEO1*). According to functional analysis (Table 3), DEGs in module A were mainly associated with cGMP-mediated signaling (*P* = 1.47E−06) and anti-apoptosis-related functions (*P* = 7.95E−06); genes in module B were mainly related to ion transport-associated functions (*P* = 1.94E−05) while DEGs in module C were most significantly involved in the regulation of Rho and Ras protein signal transduction (*P* = 3.21E−08).

**Table 3:** Functional enrichment analysis for the 16 differentially expressed genes in the three significant modules

GO-ID	*P* value	corr *P* value	*x*	Description
Module A				
19934	1.47E−06	8.16E−04	2	cGMP-mediated signaling
6916	7.95E−06	2.21E−03	3	Anti-apoptosis
48856	6.61E−05	8.68E−03	5	Anatomical structure development
43066	1.13E−04	8.68E−03	3	Negative regulation of apoptosis
43069	1.17E−04	8.68E−03	3	Negative regulation of programmed cell death
1936	1.19E−04	8.68E−03	2	Regulation of endothelial cell proliferation
51239	1.26E−04	8.68E−03	4	Regulation of multicellular organismal process
60548	1.31E−04	8.68E−03	3	Negative regulation of cell death
10646	1.49E−04	8.68E−03	4	Regulation of cell communication
32502	1.56E−04	8.68E−03	5	Developmental process
Module B				
6811	2.28E−07	1.94E−05	5	Ion transport
6813	2.82E−06	1.20E−04	3	Potassium ion transport
55085	9.85E−06	2.79E−04	4	Transmembrane transport
15672	4.56E−05	9.69E−04	3	Monovalent inorganic cation transport
51899	6.14E−05	1.01E−03	2	Membrane depolarization
6810	7.76E−05	1.01E−03	5	Transport
51234	8.34E−05	1.01E−03	5	Establishment of localization
30001	1.42E−04	1.50E−03	3	Metal ion transport
51179	1.67E−04	1.58E−03	5	Localization
6812	2.86E−04	2.43E−03	3	Cation transport
Module C				
35023	3.21E−08	7.84E−06	4	Regulation of Rho protein signal transduction
46578	6.27E−07	5.06E−05	4	Regulation of Ras protein signal transduction
6917	8.14E−07	5.06E−05	4	Induction of apoptosis
12502	8.29E−07	5.06E−05	4	Induction of programmed cell death
51056	1.58E−06	7.73E−05	4	Regulation of small GTPase-mediated signal transduction
8624	2.74E−06	1.12E−04	3	Induction of apoptosis by extracellular signals
43065	6.29E−06	1.59E−04	4	Positive regulation of apoptosis
43068	6.50E−06	1.59E−04	4	Positive regulation of programmed cell death
6915	6.64E−06	1.59E−04	4	Apoptosis
10942	7.15E−06	1.59E−04	4	Positive regulation of cell death

### Transcription regulatory network

**Fig. 3:**
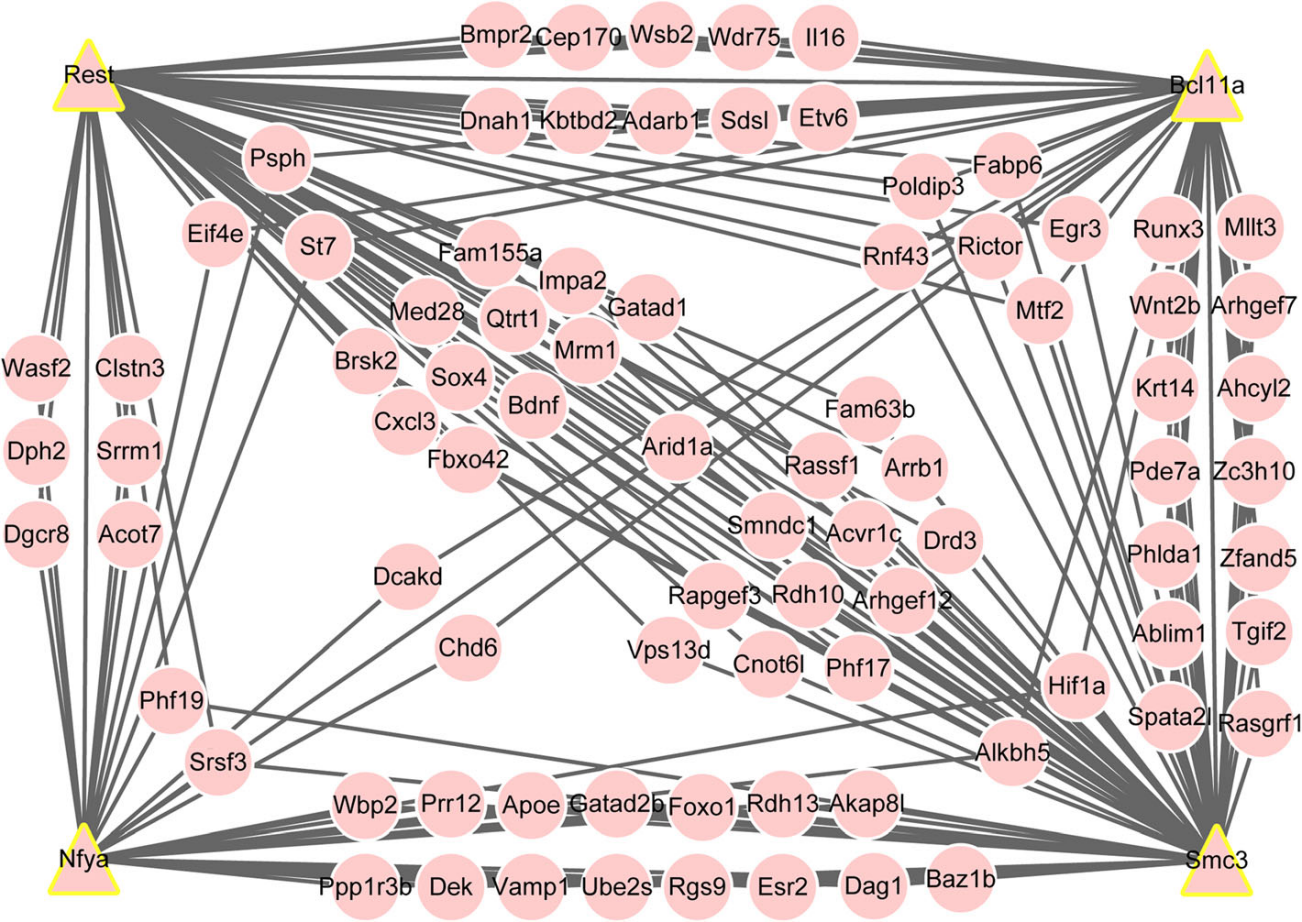
Transcription regulatory network. *Triangles* represent transcription factors and *circles* represent targeted genes

Based on information of transcription factors from ENCODE and the PPI network, 387 interacting pairs (Additional file 3: Table S3) were screened out for construction of the transcription regulatory network (Fig. 3). Analysis of functional enrichment showed that the overlapping nodes were significantly enriched in GO terms such as intracellular signaling cascade, regulation of cell proliferation, and regulation of apoptosis, among others.

## Discussion

Currently, the incidence of fractures of proximal femur have increased as industrial societies become older [23]. Femur fracture is thought to be associated with bone overgrowth, which is a common phenomenon, particularly in children. However, the underlying mechanism remains unclear. In this study, we identified potential genes involved in the molecular mechanism of bone overgrowth after femoral fracture in juvenile rats by using high-throughput bioinformatics. Based on gene expression profiles, a total of 680 DEGs were screened out, including 238 up- and 442 down-regulated DEGs. The up-regulated DEGs were found to be significantly enriched in six pathways, while down-regulated DEGs were strikingly enriched in the cancer pathway and calcium signaling pathway. PPI network construction accompanied by module detection revealed key genes such as *CCL2*, *CSF2*, *NOS2*, and *DLC1* were identified to be potentially related with femoral overgrowth.

*CCL2*, also known as monocyte chemoattractant protein-1 and small inducible cytokine A2, is a chemokine ligand and plays a crucial role in the recruitment and activation of macrophages/monocytes during inflammation after bone injury [24]. It is well known that activation of macrophages and monocytes can stimulate osteoclastic bone resorption or bone formation [25, 26]. However, the recruitment of macrophages and monocytes to the inflamed bone by CCL2 is regulated by rhTNF to regulate bone formation and further improve fracture healing, which only occurs in the fractured environment [27–29]. Additionally, the production of CCL2 is stimulated by the receptor-activator of nuclear factor (NF)-κB ligand, which is regarded as an essential regulator of bone remodeling [30]. In the present study, *CCL2* was found to be up-regulated at the proximal femoral growth plate of mid-shaft fracture samples compared with that of no fracture samples and was predicted to participate in cell functions; *CCL2* was a key node in the PPI network constructed of significant interaction pairs of DEGs. Thus, *CCL2* may be involved in bone overgrowth after femora fracture via recruiting macrophages and monocytes to fractured bone to accelerate bone formation.

*CSF2*, also known as granulocyte macrophage colony-stimulating factor (GM-CSF), encodes a monomeric glycoprotein which is regarded as a hemopoietic growth factor. CSF2 is released by osteoblast lineage cells [31]. CSF2 is associated with the production, differentiation, and function of granulocytes and macrophages in vitro [32]. The fusion of monocytes/macrophages can form osteoclasts, which were demonstrated to function in degrading bone [31, 33]. Additionally, CSF2 was confirmed to be a target of NF-κB for inducing osteoclastogenesis and further promoting osteolytic bone metastasis [34]. CSF2 has also been reported to enhance osteoclast development which was mediated by tumor necrosis factor α [35]. In addition, *CSF2* is an anti-apoptotic factor that can minimize the extent of cell death (such as osteoclasts) in tissues surrounding the injured areas [36]. In this study, *CSF2* was identified to be down-regulated. As a result, reduced *CSF2* may negatively regulate osteoclastogenesis, resulting in partial recovery of bone formation.

NOS2 is an isoenzyme of nitric oxide (NO) synthase and plays an important role in producing NO, a multifunctional signal molecule. Osteoclasts have been confirmed to express *NOS2* and release NO in a regulated manner [37–40]. In addition, NO is involved in the mechanism of osteoclastic activity by releasing bone-resorbing inflammatory cytokines [41]. Endogenously produced NO exerts potent biphasic actions that may significantly affect the proliferation, recruitment, differentiation, and/or survival of osteoblasts and osteoclasts [42–44]. Low levels of NO may support osteoblast bone formation and osteoclast-mediated bone remodeling as well as protect osteoblasts against apoptosis, while high NO levels inhibit osteoclastogenesis and prevent bone loss [45–47]. Additionally, NO is involved in the control of Ca^2+^ dynamics and mediates Ca^2+^-inhibited bone resorption [39, 48]. Furthermore, epidermal growth factor receptor/signal transducers and activators of transcription 3 can interact with the *NOS2* promoter and activate *NOS2* expression [49]. In this study, NOS2 was predicted to participate in anti-apoptosis-related functions and was found to be significantly enriched in the calcium pathway and cancer pathway. Therefore, NOS2 may participate in bone overgrowth after femora fracture via suppressing osteoblast apoptosis through the cancer pathway and calcium pathway. However, the regulation of NOS2 in bone overgrowth after fracture requires further investigation.

*DLC1* encodes a Rho GTPase-activating protein that regulates osteoclastogenesis via Rho protein signal transduction [50]. Moreover, Rho GTPases have been confirmed to play a critical role in regulating the actin cytoskeleton organization of osteoclasts [51]. In the present study, *DLC1* was found to be down-regulated and enriched in the functions of regulation of Rho protein signal transduction in module C. Thus, this gene may also be important for reducing osteoclastogenesis and bone resorption after femur fracture.

## Conclusions

The identified DEGs, particularly those in significant gene modules, including *CCL2*, *CSF2*, *NOS2*, and *DLC1*, may play a vital role in bone overgrowth after mid-shaft femur fracture. Experimental studies including samples of a larger size will be performed in the future. These data underscore the complexity of the regulation of bone overgrowth. Additionally, these findings form a basis for future studies focusing on the role of these key genes in the molecular mechanisms of bone growth disturbances with the longer-term goal of investigating proper treatment for children with fractured growing bones.

## Additional files

Additional file 1: All the DEGs screened between intact and fractured samples. (XLS 96 kb)
Additional file 2: Significant protein interactions in the PPI network.ᅟ(XLS 39 kb)
Additional file 3: The interacting pairs used for construction of the transcription regulatory network.ᅟ(XLS 24 kb)

## Abbreviations

CCL2: Chemokine (C-C motif) ligand 2
CSF2: Colony-stimulating factor 2
DEGs: Differentially expressed genes
ENCyclopedia of DNA Elements: The ENCODE
FGFR: Fibroblast growth factor receptor
GM-CSF: Granulocyte macrophage colony-stimulating factor
GO: Gene ontology
LRP4: Lipoprotein receptor-related protein 4
MCODE: Molecular complex detection
NO: Nitric oxide
NOS2: Nitric oxide synthase 2
PPI: Protein-protein interaction
